# Modeling the adenosine system as a modulator of cognitive performance and sleep patterns during sleep restriction and recovery

**DOI:** 10.1371/journal.pcbi.1005759

**Published:** 2017-10-26

**Authors:** Andrew J. K. Phillips, Elizabeth B. Klerman, James P. Butler

**Affiliations:** 1 Division of Sleep Medicine, Departments of Medicine and Neurology, Harvard Medical School, and Division of Sleep and Circadian Disorders, Brigham and Women's Hospital, Boston, MA, United States of America; 2 Division of Pulmonary and Critical Care Medicine, Brigham and Women’s Hospital, and Department of Medicine, Harvard Medical School, Boston, MA, United States of America; 3 Department of Environmental Health, Harvard T.H. Chan School of Public Health, Boston, MA, United States of America; Human Sleep Psychopharmacology Laboratory, Institute of Pharmacology & Toxicology, UNITED STATES

## Abstract

Sleep loss causes profound cognitive impairments and increases the concentrations of adenosine and adenosine A_1_ receptors in specific regions of the brain. Time courses for performance impairment and recovery differ between acute and chronic sleep loss, but the physiological basis for these time courses is unknown. Adenosine has been implicated in pathways that generate sleepiness and cognitive impairments, but existing mathematical models of sleep and cognitive performance do not explicitly include adenosine. Here, we developed a novel receptor-ligand model of the adenosine system to test the hypothesis that changes in both adenosine and A_1_ receptor concentrations can capture changes in cognitive performance during acute sleep deprivation (one prolonged wake episode), chronic sleep restriction (multiple nights with insufficient sleep), and subsequent recovery. Parameter values were estimated using biochemical data and reaction time performance on the psychomotor vigilance test (PVT). The model closely fit group-average PVT data during acute sleep deprivation, chronic sleep restriction, and recovery. We tested the model’s ability to reproduce timing and duration of sleep in a separate experiment where individuals were permitted to sleep for up to 14 hours per day for 28 days. The model accurately reproduced these data, and also correctly predicted the possible emergence of a split sleep pattern (two distinct sleep episodes) under these experimental conditions. Our findings provide a physiologically plausible explanation for observed changes in cognitive performance and sleep during sleep loss and recovery, as well as a new approach for predicting sleep and cognitive performance under planned schedules.

## Author summary

Sleep loss is known to cause significant decrements in cognitive performance, but the physiological mechanisms responsible for this response are not well understood. Computational models have been developed to predict how individuals will cognitively perform under acute or chronic sleep loss, but they currently lack an explicit physiological foundation, and do not specifically predict sleep timing. Adenosine is hypothesized to be an important mediator in the effects of sleep loss, as it is a sleep-promoting substance that accumulates in the brain during wakefulness. We developed a mathematical model of the adenosine system in the brain and showed that it can parsimoniously account for not only changes in cognitive performance during acute sleep deprivation, chronic sleep restriction, and recovery, but also changes in sleep patterns during long-term recovery. The model thus provides a quantitative link between complex whole-organism behaviors and underlying molecular and physiologic mechanisms.

## Introduction

When sleep is restricted, cognitive performance declines, recovering again when adequate sleep is obtained. The dynamics of performance decline and recovery depend on the timescales over which sleep loss occurs. During 1–2 nights of sleep deprivation (continuous wakefulness), cognitive performance declines rapidly, and then returns to baseline after 1–2 nights of recovery sleep [1,2]. However, when sleep restriction is chronic (i.e., multiple nights of insufficient sleep), such as 1–2 weeks of 3–6 hours of sleep per night, performance declines steadily on a timescale of weeks or longer [3,4], and performance remains significantly impaired even after 2–3 nights of recovery sleep [5].

Mathematical models have been developed to describe the effects of different sleep schedules on physiology and cognitive performance. In 1984, Daan et al. [6] developed a mathematical model, called the “two-process model”, to describe the effects of regular sleep schedules and sleep deprivation on EEG slow-wave activity, which is one marker of “sleep debt”. The two-process model assumes that sleep is regulated by two independent processes: a circadian process, which describes the approximately 24-hour rhythm in sleepiness, and the sleep homeostatic process, which describes the tendency to accrue sleep debt and become sleepier the longer one is awake. The dynamics of the sleep homeostatic process consist of exponential saturation towards an upper threshold during wake, with a time constant of ~20 h, and exponential decay towards a lower threshold during sleep, with a time constant of ~4 h in young adults. Variants of the two-process model have also been used to describe changes in cognitive performance with sleep loss [7–12]. This whole family of models, however, fails to describe the long-timescale changes in cognitive performance that occur under chronic sleep restriction [3,5,13,14]. This is because the models lack any time constants longer than ~20 h, so the effects of any particular sleep regime (restriction or recovery) rapidly saturate.

To address this problem, extensions of the two-process model were developed [15–18]. These models include an additional long-timescale process that modulates the upper and/or lower saturation thresholds for the sleep homeostatic process. Adding this additional degree of freedom allows the models to capture changes in cognitive performance under both acute sleep deprivation and chronic sleep restriction. These long-timescale model processes are, however, ad hoc, and not based on physiology. Additional insights and opportunities for intervention design could be gained if these models were based on the physiological processes that underlie the sleep homeostatic process, including the adenosine system in particular.

Sleep-promoting substances, including adenosine, accumulate in multiple regions of the brain during wakefulness [19]. In addition, adenosine A_1_ receptors in the brain are up-regulated by sleep loss [20,21]. McCauley et al. [17] noted that the dynamics of their model “could be a mathematical representation of the interaction between a neurotransmitter or neuromodulator and its receptor, with the density of both changing dynamically across time awake and time asleep”; they identified the adenosine system as a probable candidate [22]. However, no explicit link was made between their model and the underlying physiology; we show below that their model’s dynamical structure differs from our model of the adenosine system.

Understanding the physiological basis for cognitive impairments associated with sleep restriction is important, given that approximately 30% of the adult US population sleeps less than 7 hours per night, which is below the 7–9 hour range recommended by the National Sleep Foundation [23]. Moreover, impaired cognition due to sleep loss is associated with errors and accidents [24]. Here, we develop an explicit mathematical model of the adenosine system, with the goal of testing the hypothesis that dynamic changes in the concentrations of both adenosine molecules and receptors can account for changes in cognitive performance and sleep patterns under acute sleep deprivation, chronic sleep restriction, and recovery from chronic sleep restriction. Our model is tested against two previously published experimental data sets: (i) psychomotor vigilance test (PVT) data during acute and chronic sleep restriction, and recovery from chronic sleep restriction; and (ii) sleep durations during long sleep opportunities in individuals recovering from low-level chronic sleep restriction.

## Materials and methods

**Fig 1:**
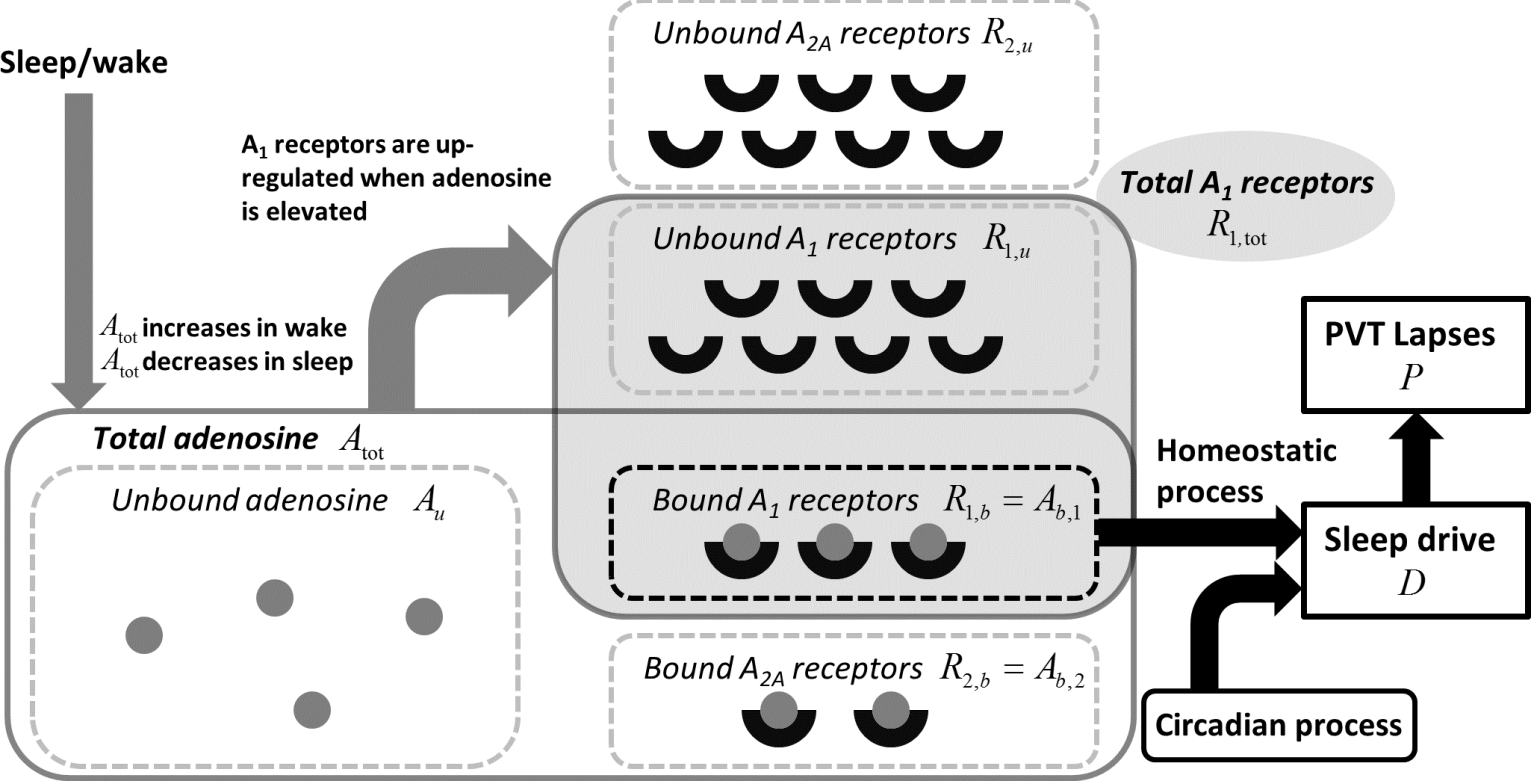
Schematic of the mathematical model. Sleep/wake state determines the dynamics of the total adenosine concentration, *A*_tot_, with *A*_tot_ increasing in wake and decreasing in sleep. This pool fractionates into the concentrations of those unbound, *A*_*u*_, those that are bound to A_1_ receptors, *A*_1,*b*_, and those that are bound to A_2A_ receptors, *A*_2,*b*_. The model also includes pools of A_1_ and A_2A_ receptors, with total concentrations of *R*_*n*,tot_, n = 1 or 2, which fractionate into bound, *R*_*n*,*b*_, and unbound *R*_*n*,*u*_,. The total concentration of A_1_ receptors, *R*_1,tot_ = *R*_1,*b*_ + *R*_1,*u*_, changes in response to adenosine concentration by attempting to achieve a homeostatic level of fractional occupancy, *R*_1,*b*_/*R*_1,tot_. For modeling sleep and performance, *R*_1,*b*_ is used as the sleep homeostatic process. This is added to a circadian process to give the overall sleep drive, *D*. A sigmoid function is then used to convert *D* into PVT lapses, *P*.

We first develop a pharmacokinetic model of the adenosine system, including the dynamics of the concentrations of adenosine molecules and adenosine receptors. We use this model to give a new physiological definition of the sleep homeostatic process, and then link this process to performance on the psychomotor vigilance test (PVT). Methods used to estimate parameter values are then described. A schematic of the model and its variables is shown in **Fig 1**.

### The adenosine system

Extracellular adenosine concentration increases during wakefulness and decreases during sleep in multiple brain regions, including the basal forebrain where its action is important to sleep regulation [25]. Time-courses for increase and decrease of adenosine concentration have not been precisely characterized, but we can make reasonable physiological assumptions; see e.g., [26]. Specifically, we assume adenosine is produced at a constant rate in wakefulness and at a constant (but lower) rate in sleep, due to the lower (on average) brain metabolism during sleep [27]. We also assume that adenosine follows first-order pharmacokinetics, i.e., it is cleared at a rate proportional to its concentration in both wake and sleep, with clearance being faster during sleep due to active removal of metabolites [28]. Adenosine concentrations can vary between different brain regions [19]; here we calibrate the model against data collected from the basal forebrain, given its important role in sleep regulation [25]. We do not model regional differences in concentrations within the brain.

These assumptions yield the following ordinary differential equation for total adenosine concentration,

$$\chi \frac{dA_{\mathrm{tot}}}{dt}=\mu -A_{\mathrm{tot}}.$$ (1)

The solution of this is exponential towards the saturation value *μ* with a time constant *χ*. The values of *χ* and *μ* depend on sleep/wake state, which is a binary input to the model (i.e., the model can be either awake or asleep at any given time). Across sleep/wake transitions, we demand continuity of *A*_tot_.

Total adenosine concentration includes: concentration of unbound molecules, denoted *A*_*u*_; concentration of molecules bound to A_1_ receptors, denoted *A*_1,*b*_; and concentration of molecules bound to A_2A_ receptors, denoted *A*_2,*b*_. We do not consider A_2B_ or A_3_ receptors here, due to their much lower affinity for adenosine [29]. Thus,

$$A_{\mathrm{tot}}=A_{u}+A_{1,b}+A_{2,b}.$$ (2)

Concentrations of the different pools of adenosine depend on the availability of A_1_ and A_2A_ receptors. For receptor type *n* (where *n* is 1 or 2A, abbreviated by 2), we denote total receptor concentration by *R*_n,tot_. Total receptor concentration includes: concentration of unbound receptors, denoted *R*_*n*,*u*_; and concentration of bound (occupied) receptors, denoted *R*_*n*,*b*_, which by definition equals *A*_*n*,*b*_. Thus,

$$R_{1,\mathrm{tot}}=R_{1,u}+R_{1,b},$$ (3)

$$R_{2,\mathrm{tot}}=R_{2,u}+R_{2,b}.$$ (4)

We use mass-action kinetics to describe the rates of binding and unbinding at each receptor type,

$$\frac{dA_{u}}{dt}=-k_{1,b}A_{u}R_{1,u}-k_{2,b}A_{u}R_{2,u}+k_{1,u}R_{1,b}+k_{2,u}R_{2,b},$$ (5)

$$\frac{dR_{1,b}}{dt}=k_{1,b}A_{u}R_{1,u}-k_{1,u}R_{1,b},$$ (6)

$$\frac{dR_{2,b}}{dt}=k_{2,b}A_{u}R_{2,u}-k_{2,u}R_{2,b}.$$ (7)

Parameters *k*_*n*,*b*_ and *k*_*n*,*u*_ are rate constants for binding and unbinding at receptor type *n*, respectively. The equilibrium conditions for Eqs (5)–(7) can be written in terms of ratios of rate constants,

$$K_{d1}=\frac{k_{1,u}}{k_{1,b}}=\frac{A_{u}R_{1,u}}{R_{1,b}},$$ (8)

$$K_{d2}=\frac{k_{2,u}}{k_{2,b}}=\frac{A_{u}R_{2,u}}{R_{2,b}}.$$ (9)

These ratios are the dissociation constants for each reaction and have units of concentration. The value of *K*_*dn*_ can be interpreted as the concentration of unbound adenosine, *A*_*u*_, for which there are equal numbers of bound and unbound receptors: *R*_*n*,*b*_ = *R*_*n*,*u*_.

It has been experimentally observed that the concentration of A_1_ receptors increases when sleep is restricted and adenosine concentrations are elevated, and decreases to normal following recovery [30]. We hypothesize here that this phenomenon reflects the dynamics of a (homeostatic) cellular response to maintain stable levels of A_1_ receptor occupancy. When adenosine levels are elevated, a greater fraction of A_1_ receptors will be occupied. To return to a homeostatic level of occupancy, more receptors must be synthesized. This is a physiologically reasonable hypothesis, as receptor occupancy rates could be sensed and integrated on a per-cell basis. We model these dynamics as:

$$\lambda \frac{dR_{1,\mathrm{tot}}}{dt}=R_{1,b}-\gamma R_{1,\mathrm{tot}},$$ (10)

where 0 < *γ* < 1 is the target occupancy fraction, with equilibrium at *R*_1,*b*_/*R*_1,tot_ = *γ*, and *λ* is a time constant that determines how quickly receptors are up-regulated or down-regulated in response to a change in occupancy.

We assume that *R*_2,tot_ is fixed, because only A_1_ receptors have been convincingly demonstrated to up-regulate in response to chronic sleep restriction [30].

The evolution of *A*_tot_ and *R*_1,tot_ occurs on the timescale of hours to weeks, which is much slower than the chemical rate constants. The system can therefore be timescale separated by assuming Eqs (5)-(7) are at equilibrium (i.e., they are quasistatic). We can then solve Eqs (8) and (9) for *A*_1,*b*_ and *A*_2,*b*_ in terms of *A*_tot_, *R*_1,tot_, and *R*_2,u_ (the concentration of unbound A_2A_ receptors, which can be assumed to be approximately constant and is estimated below),

$$A_{1,b}=\frac{1}{2}\left[A_{\mathrm{tot}}+R_{1,\mathrm{tot}}+\frac{K_{d1}}{1-\beta }-\sqrt{{\left(A_{\mathrm{tot}}+R_{1,\mathrm{tot}}+\frac{K_{d1}}{1-\beta }\right)}^{2}-4A_{\mathrm{tot}}R_{1,\mathrm{tot}}}\right]$$ (11)

$$A_{2,b}=\beta (A_{\mathrm{tot}}-A_{1,b}),$$ (12)

where

$$\beta =\frac{R_{2,u}}{R_{2,u}+K_{d2}},$$ (13)

Substituting Eq (11) into Eq (10) gives a closed two-dimensional system for *A*_tot_ and *R*_1,tot_ that depends on the values of *K*_*d*1_ and *K*_*d*2_, which are known from experiment, and does not depend on the values of the individual *k*_*n*,*b*_ and *k*_*n*,*u*_ parameters.

### Cognitive performance and sleep

Human cognitive performance and sleep depend on both the circadian process and the sleep homeostatic process [6]. Here, we model the sleep homeostatic process as the concentration of bound A_1_ adenosine receptors, *R*_1,*b*_. In this respect, our model differs from previous models, which have usually treated the sleep homeostatic process per se as a predictor for EEG slow wave activity or cognitive performance. We choose bound receptor concentration as the relevant sleep homeostatic variable over total receptor concentration or total adenosine concentration, because it is the downstream consequence of binding that mediates physiological effects. The sleep homeostatic process could also depend on *R*_2,*b*_, as well as other sleep-promoting molecules and receptors, such as cytokines [31], but we choose the adenosine model here as the most parsimonious basis for a computational model, and first probe its explanatory power.

We model the circadian process by a sinusoid with a period of 24 hours. This is a reasonable assumption for individuals who are entrained to the 24-hour day during chronic sleep restriction or recovery, or for individuals who are undergoing a brief acute sleep deprivation under constant conditions [32], which are the experimental conditions modeled here. Under more complicated scenarios, such as shifting time-zones, a dynamic circadian model should be used to account for changes in amplitude and phase [33].

We assume that the overall influence of the circadian and homeostatic processes on sleep and performance is represented by a linear combination of the two processes, which we call the overall sleep drive,

$$D=R_{1,b}+a\;\cos\;\omega (t-\phi ),$$ (14)

where *a* > 0 is the circadian amplitude, *ω* = (2*π*/24)h^-1^, and *ϕ* is the clock-time (modulo 24, where zero is midnight) at which the circadian process maximally promotes sleep. This is typically near the core body temperature minimum in the early hours of the morning [33]. Using *D*, we can predict when the model is sleepy (high values of *D*) or alert (low values of *D*).

There is evidence of nonlinear interactions between the circadian and homeostatic processes [34,35], and these have been introduced in some models [8,36]. Here, we choose to start with the linear form, with the possibility of extending the model in future.

As a metric for cognitive performance, we use the number of lapses (defined as responses slower than 500ms) on the 10-minute PVT task. This metric is bounded. It is not possible to have fewer than 0 lapses, and the length of the test imposes a maximum possible number of lapses. Thus, we choose a sigmoid function for converting *D* into an estimate of PVT lapses,

$$P=\frac{p_{\max}}{1+e^{\left(\frac{D_{\mathrm{mid}}-D}{D_{s}}\right)}},$$ (15)

where *p*_max_ is the maximum possible number of lapses, *D*_mid_ is the value of *D* for which the half-maximum value of lapses is achieved, and *D*_*s*_ determines the width of the sigmoid. The variables *D* and *P* are both continuous functions of time. For comparison with experiments, we sample *P* at times when a PVT test was performed.

### Approach to fitting

In this section, we describe how the model parameters are fit and how the model outputs are compared to experimental data. We first estimate physiological ranges for a subset of the model parameters and the mathematical relationships between others. Due to the nature of the model and data, we then fit all the parameter values using an iterative approach. For Experiment 1, the dependent variable is PVT lapses, and sleep/wake timing is given as an input to the model via Eq (1). Values of all model parameters are fit at this stage, with the exception of *λ*, which takes a nominal value. This is valid due to the fact that the model predictions for Experiment 1 are only weakly dependent on *λ*. The model is then applied to simulate Experiment 2. For this experiment, the dependent variable is daily sleep duration. Two new parameters, *D*_sleep_, and *D*_wake_, are introduced to allow the model to make automatic transitions between sleep and wake (i.e., the model no longer requires sleep/wake timing as an input). The values of the three parameters *λ*, *D*_sleep_, and *D*_wake_ are thus fit to Experiment 2, with all other parameters taking the values previously obtained by fitting to Experiment 1. Finally, the values of *D*_mid_ and *D*_*s*_ are recalibrated to ensure the model still optimally fits data from Experiment 1. Details of this fitting procedure are provided below.

### Parameter estimation

In the adenosine system model, some parameters can be estimated from existing data. The dissociation constants for *K*_*d*1_ and *K*_*d*2_ for A_1_ and A_2A_ receptors are 1-10nM and 100–10,000nM, respectively [37]. The fact that *K*_*d*1_ ≈ *K*_*d*2_/100 means that A_1_ receptors have much higher affinity for binding adenosine molecules (i.e., equivalent binding at 1/100 the concentration).

The time constants in Eqs (1) and (10) can be estimated based on the time-course of sleep homeostasis. The hypothesis we wish to test with our model is that variations in *A*_tot_ on timescales of hours to days can account for short timescale variations in sleep homeostatic pressure such as acute sleep deprivation, whereas variations in *R*_1,tot_ on timescales of weeks to months can account for long timescale effects of chronic sleep restriction. The value of *λ* must therefore be large. The longest inpatient chronic sleep restriction experiment to date lasted 3 weeks, with large decreases in PVT performance between weeks 1 and 2, followed by non-significant decreases in PVT performance between weeks 2 and 3 [3]. This suggests *λ* is on the order of 1–2 weeks. The dynamics of the model in Experiment 1 are found to be relatively insensitive to the value of *λ*, so we use a nominal value of 300 h (the fit value ends up being close to this initial guess). The value of *λ* is then refined by fitting the model to Experiment 2.

Since *R*_1,tot_ is slowly varying, it can be considered approximately constant on a timescale of hours or shorter. On this timescale, only *A*_tot_ significantly varies, so the dynamics of sleep homeostatic pressure described by the two-process model should approximately correspond to the dynamics of *A*_tot_ described by Eq (1). Thus, we use the time constants of the original two-process model, *χ*_wake_ = 18.18 h and *χ*_sleep_ = 4.20 h [6].

Biochemical data also give us typical values for the concentrations *R*_1,tot_, *R*_2,tot_, and *A*_*u*_, which can be used to establish approximate quantitative relationships between some of the model’s parameters. In the human cerebral cortex, *R*_1,tot_, is approximately 600nM, and *R*_2,tot_, is approximately 300nM, with some variation in both between brain regions [38]. In mammals, microdialysis measurements of extracellular unbound adenosine concentration, *A*_*u*_, report concentrations around 30nM [19]. Since the dissociation constant for A_2A_ receptors is large compared to physiological concentrations of *A*_*u*_, it is reasonable to assume *R*_2,*u*_ ≈ *R*_2,tot_ in Eq (13).

In an individual who is well rested (i.e., not sleep restricted) and keeping a regular daily sleep/wake cycle, *A*_*u*_ will make daily oscillations about a stable level in response to sleep/wake cycles, causing daily oscillations in *R*_1,*b*_ = *A*_*b*,1_, following the relationship described in Eq (11). In steady state, Eq (10) can be written <*R*_1,*b*_> = *γ*(<*R*_1,*b*_> + <*R*_1,*u*_>), where <∙> denotes expected value, and Eq (8) (valid only at equilibrium) can be rewritten in term of its time average as $<R_{1,b}>=K_{d1}^{-1}<A_{u}><R_{1,u}>[1+h.o.]$, where *h*.*o*. denotes the time average of higher order terms (multiplicative cross products). Combining these yields:

$$\gamma \approx \frac{\langle A_{u}\rangle }{\langle A_{u}\rangle +K_{d1}}.$$ (16)

Given *A*_*u*_ is typically around 30nM and *K*_*d*1_ is 1-10nM, this gives an estimated value of 0.50–0.91 for *γ*.

Using Eqs (2) and (11)–(13), the parameters *A*_tot_, *A*_*u*_, *K*_*d*1_, and *β* are related by

$$2A_{u}=(1-\beta )\left(A_{\mathrm{tot}}-R_{1,\mathrm{tot}}-\frac{K_{d1}}{1-\beta }+\sqrt{{\left(A_{\mathrm{tot}}+R_{1,\mathrm{tot}}+\frac{K_{d1}}{1-\beta }\right)}^{2}-4A_{\mathrm{tot}}R_{1,\mathrm{tot}}}\right)$$ (17)

Rearranging for *A*_tot_ gives

$$A_{\mathrm{tot}}=\frac{A_{u}(A_{u}+K_{d1}+R_{1,\mathrm{tot}}(1-\beta ))}{\left(A_{u}+K_{d1})(1-\beta \right)}.$$ (18)

Given values of *K*_*d*1_ and *β*, we use the typical value of *A*_*u*_ ≈ 30nM to estimate a typical value of *A*_tot_. Finally, we relate the value of *A*_tot_ to the parameters *μ*_wake_ and *μ*_sleep_ in Eq (1). During wake, the solution of Eq (1) as a function of time into the wake episode is

$$A_{\mathrm{tot},\mathrm{wake}}(t)=\mu_{\mathrm{wake}}+(A_{\mathrm{tot},\mathrm{wake}}(0)-\mu_{\mathrm{wake}})e^{-t/\chi_{\mathrm{wake}}}.$$ (19)

Similarly, during sleep, the solution of Eq (1) as a function of time into the sleep episode is

$$A_{\mathrm{tot},\mathrm{sleep}}(t)=\mu_{\mathrm{sleep}}+(A_{\mathrm{tot},\mathrm{sleep}}(0)-\mu_{\mathrm{sleep}})e^{-t/\chi_{\mathrm{sleep}}}.$$ (20)

For an individual who keeps a regular 24-hour sleep/wake cycle with a block of *T* hours of sleep per day, continuity of Eqs (19) and (20) require that

$$A_{\mathrm{tot},\mathrm{wake}}(24-T)=A_{\mathrm{tot},\mathrm{sleep}}(0),$$ (21)

$$A_{\mathrm{tot},\mathrm{sleep}}(T)=A_{\mathrm{tot},\mathrm{wake}}(0).$$ (22)

Combining these conditions gives the initial values of *A*_tot_ at the beginning of each sleep and wake episode, respectively,

$$A_{\mathrm{tot},\mathrm{sleep}}(0)=\frac{\mu_{\mathrm{wake}}(1-e^{\frac{T-24}{\chi_{\mathrm{wake}}}})+\mu_{\mathrm{sleep}}(1-e^{\frac{-T}{\chi_{\mathrm{sleep}}}})e^{\frac{T-24}{\chi_{\mathrm{wake}}}}}{1-e^{\frac{T-24}{\chi_{\mathrm{wake}}}-\frac{T}{\chi_{\mathrm{sleep}}}}},$$ (23)

$$A_{\mathrm{tot},\mathrm{wake}}(0)=\frac{\mu_{\mathrm{sleep}}(1-e^{\frac{-T}{\chi_{\mathrm{sleep}}}})+\mu_{\mathrm{wake}}(1-e^{\frac{T-24}{\chi_{\mathrm{wake}}}})e^{\frac{-T}{\chi_{\mathrm{sleep}}}}}{1-e^{\frac{T-24}{\chi_{\mathrm{wake}}}-\frac{T}{\chi_{\mathrm{sleep}}}}}.$$ (24)

For a human with a typical schedule (*T* = 8 h), substituting the numerical values of *χ*_wake_ and *χ*_sleep_ in Eqs (21) and (22), and averaging these, we obtain an approximate estimate of a typical total adenosine concentration in the model,

$$A_{\mathrm{tot}}=0.36\mu_{\mathrm{wake}}+0.65\mu_{\mathrm{sleep}}.$$ (25)

Given values of *K*_*d*1_ and *K*_*d*2_ within their respective physiological ranges, we use Eq (18) to estimate *A*_tot_. For each value of *A*_tot_ we then obtain a range of values for *μ*_wake_ and *μ*_sleep_ using Eq (25). We require *μ*_wake_ > *μ*_sleep_ > 0.

In the performance model described in Eq (15), the parameter *p*_max_ can also be estimated. In the standard 10-minute PVT, trials occur every 2–10 seconds. Inter-trial intervals are drawn uniformly randomly from this time interval, with an average inter-trial interval of 6 seconds. For a typical response time of *δ*, the number of trials per PVT is 600/(6 + *δ*). The theoretical maximum number of lapses would occur in an individual who responded in exactly 500ms on each trial, giving 92 lapses. In reality, lapses are often much longer than 500ms [39]. Individuals subject to a combination of acute sleep deprivation and severe chronic sleep restriction approach 4 seconds as a median response time [3]. This suggests a theoretical ceiling of *p*_max_ ≈ 60 lapses.

### Fitting model parameters for Experiment 1

The first test of the model is whether it can account for PVT data collected in humans undergoing acute sleep deprivation or different levels of chronic sleep restriction. In Experiment 1 four groups of healthy young adults were exposed to different conditions of sleep restriction [4]. One group (n = 13) underwent acute sleep deprivation for 88 h. The other groups underwent chronic sleep restriction for 13 nights (4 h time in bed per night for n = 13, 6 h time in bed per night for n = 13, and 8 h time in bed per night for n = 9), followed by 2 recovery nights (8 h time in bed per night). Average sleep times per night during the chronic sleep restriction were approximately 3.7 h, 5.5 h, and 6.8 h, for the 4 h, 6 h, and 8 h time in bed conditions, respectively. In each condition, participants awoke at the same time of 7:30am, which we plotted as 8am for convenience. Prior to beginning the experiment, all four groups had three baseline nights with 8 h time in bed. In the 5 nights prior to entering the laboratory, participants reported getting an average of 7.8 h sleep per night.

During wakefulness, participants completed 10-minute PVT tests every 2 hours. Group-average PVT lapses were reported for each experimental condition in McCauley et al. [17], beginning 4 hours after awakening each day to avoid effects of sleep inertia on performance. We used these data (recorded manually from the previous paper) as our performance metric, *P*. The same data set was used previously to develop a model of the effects of chronic sleep restriction on human performance [17] and a similar data set [5] was used to develop another model [18]. It is therefore an important first test of our physiological model.

Model parameter values were chosen within the estimated ranges given in Table 1 to achieve a least-squares fit to the experimental data. This optimization was performed numerically using the Levenberg-Marquardt algorithm for global convergence. The implementation used was the nlinfit function in Matlab (version R2014A, Natick MA, USA). The optimization was initialized using parameter values that fell within physiological ranges.

**Table 1:** Fit values and units for the 15 model parameters.

Parameter	Constrained range	PVT Fit	Full Fit	Units
*K*_*d*1_	1–10	1	1	nM
*K*_*d*2_	100–10,000	100	100	nM
*χ*_wake_	18.18	18.18	18.18	h
*χ*_sleep_	4.20	4.20	4.20	h
*p*_max_	60	60	60	lapses
*D*_mid_	None	579.3	583.2	nM
*D*_*s*_	None	5.603	5.872	nM
*a*	> 0	3.25	3.25	nM
*ϕ*	0–24	7.95	7.95	h
*γ*	0.75–0.97, Eq (16)	0.9677	0.9677	1
*μ*_wake_	> *μ*_sleep_, Eq (25)	869.5	869.5	nM
*μ*_sleep_	Eq (27)	596.4	596.4	nM
*λ*	>100	300	291	h
*D*_wake_	None	N/A	555.4	nM
*D*_sleep_	None	N/A	572.7	nM

Constrained ranges exist for some parameters. In cases where equations relate a parameter to other known variables, this is given next to the constrained range. Values for the parameters *K*_*d*1_ through to *μ*_sleep_ were first fit to Experiment 1, using a nominal value of *λ* = 300 h. This yields the parameter values in the “PVT Fit” column. The last three parameters in the table were then fit to Experiment 2, and finally *D*_mid_ and *D*_*s*_ were recalibrated against Experiment 1 to yield the parameter values in the “Final Fit” column.

The model was initialized by simulating a schedule with 7.8 h sleep, matching the sleep duration participants reported getting prior to the inpatient schedule. This schedule was repeated until convergence to a limit cycle was achieved. Three baseline nights were then simulated with 7.0 h sleep per night, matching the average sleep duration participants achieved during baseline inpatient conditions. Actual sleep durations were then simulated for each condition. For consistency between all conditions, we chose to simulate recovery nights in the same manner as baseline nights, with 7.0 h sleep per night. Morning awakenings are all plotted as occurring at 8am.

For reference, we also plotted the predictions of the McCauley et al. model. For these, we used the published equations and initial conditions.

### Fitting model parameters for Experiment 2

In Experiment 2, 16 healthy young adults lived under “long night” conditions for 28 days [40]. During these days, they were required to spend 14 hours per night (6pm to 8am) in bed in a completely dark room, with no activities allowed, besides using the bathroom. Participants were free to sleep for as much of this time as they liked, and sleep was recorded with polysomnography. In the week prior to this, the participants were given 8 h time in bed per night, beginning around midnight. During this week, they averaged approximately 7 h total sleep per night, and thus likely had some residual sleep debt. While it was not primarily designed for this purpose, the experiment can be viewed as a long-term recovery from chronic low-level sleep restriction. This is extremely valuable, since most chronic sleep restriction experiments have involved a week or less of recovery, making it difficult to determine the timescale of recovery. The experimental data are strongly suggestive of a slow recovery process from an accrued sleep dept; individuals slept an average of 10.3 h across nights 1–3, 9.1 h across nights 4–7, 8.7 h across nights 8–14, 8.7 h across nights 15–21, and 8.2 h across nights 22–28. Interestingly, some individuals developed “split” sleep patterns, in which they had two main nighttime sleep bouts with a period of awakening in the middle. This finding has been used as empirical support for the historical claim that humans in pre-industrial times had split sleep patterns [41].

The estimation and fitting methods described above for Experiment 1 provide values for all parameters of the adenosine model, except *λ*. The length of Experiment 2 allows us to accurately fit the value of this parameter. In addition, we introduce two new parameters, *D*_sleep_, and *D*_wake_ that allow the model to generate its own sleep/wake patterns. During times when sleep is allowed by the schedule, the following rules are used to determine sleep/wake transitions,

$$\left\{ \begin{array}{c} D>D_{\mathrm{sleep}}:\;\mathrm{Transition}\;\mathrm{to}\;\mathrm{sleep}\;\mathrm{if}\;\mathrm{currently}\;\mathrm{awake}\\ D<D_{\mathrm{wake}}:\;\mathrm{Transition}\;\mathrm{to}\;\mathrm{wake}\;\mathrm{if}\;\mathrm{currently}\;\mathrm{asleep} \end{array}\right.$$

This is motivated by the two thresholds used for sleep/wake transitions in the two-process model [6].

The values of *λ*, *D*_sleep_, and *D*_wake_ were estimated by least-squares fitting the model’s daily total sleep durations to the experimental group-average daily total sleep durations for days 1–28 of Experiment 2. The Levenberg-Marquardt algorithm was found to perform poorly in this application, due to many points in parameter space achieving similarly good fits to the data. We therefore finely gridded parameter space to find the optimal values of *λ*, *D*_sleep_, and *D*_wake_, each to at least 3 significant figures.

The model was initialized by simulating a schedule with 7 h sleep per day, beginning at midnight. This schedule was repeated until convergence to a limit cycle was achieved. The experimental protocol was then simulated by allowing sleep between 6pm and 8am each night. More specifically, the model was forced to be awake from 8am to 6pm each day, and then freely selected sleep and wake times in the interval between 6pm and 8am each night using the thresholds described above. These conditions were maintained for 50 days, allowing the model’s behavior in the first 28 days to be compared to data and allowing us to observe the model’s predicted longer-term behavior.

Finally, the parameters *D*_mid_ and *D*_*s*_ were recalibrated against Experiment 1 data to yield their final values, resulting in modest changes in both parameters. The Levenberg-Marquardt algorithm was again used. This recalibration was necessary, because the values of *λ*, *D*_sleep_ and *D*_wake_ determine the model’s natural sleep duration during baseline and the level of initial sleep homeostatic pressure. The PVT function parameters must therefore be adjusted to allow the model to still optimally fit Experiment 1, while maintaining the same outputs for Experiment 2 (because the PVT function parameters do not affect sleep/wake outputs). All other parameters therefore remained fixed at their previously fit values.

## Results

**Fig 2:**
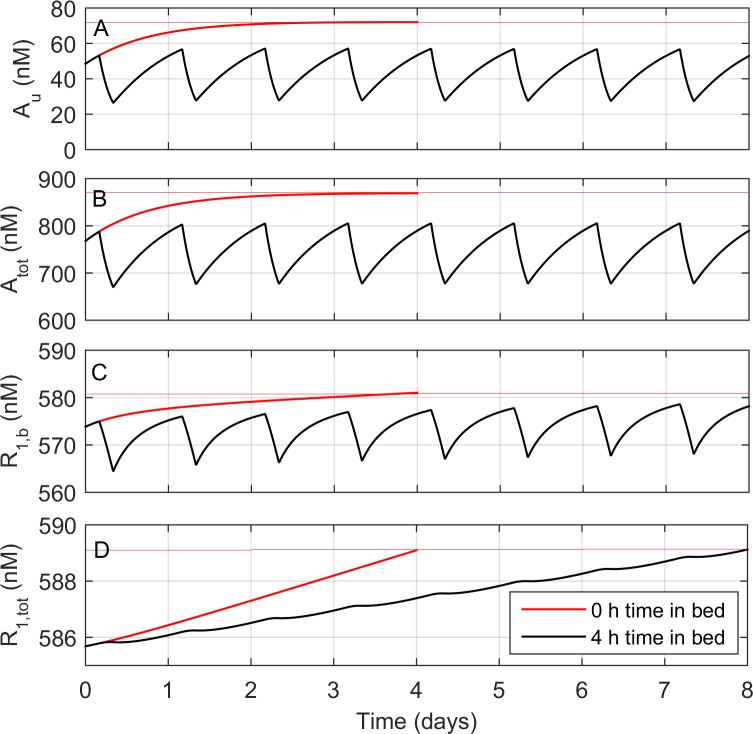
Dynamics of the adenosine model under two simulated conditions: Acute sleep deprivation for 4 days (red line), and chronic sleep restriction with 4 h sleep per night for 8 days (black line). Four model variables are shown as functions of time: (A) Unbound adenosine concentration, *A*_*u*_. (B) Total adenosine concentration, *A*_tot_. (C) Bound A_1_ receptor concentration, *R*_1,*b*_, which is used as a proxy for sleep homeostatic pressure in the model. (D) Total A_1_ receptor concentration, *R*_1,tot_. Red horizontal lines represent the level of each variable at the end of the 4 days of acute sleep deprivation.

Values for all parameters except *λ*, *D*_sleep_, and *D*_wake_ were first found by fitting the model to PVT data in Experiment 1 (**Table 1**); the three other parameters were then fit using Experiment 2, as described above. Finally, *D*_mid_ and *D*_*s*_ were recalibrated against Experiment 1, resulting in modest changes in both parameter values (**Table 1**). To illustrate the model’s essential dynamics, we show the time evolution of adenosine concentrations (unbound and total) and receptor concentrations (bound and total) in **Fig 2** for two simulated experimental conditions: 4 days of acute sleep deprivation and 8 days of chronic sleep restriction with 4 hours time in bed per night. These two conditions are chosen because they affect the sleep homeostatic process in different ways. Under acute sleep deprivation, the model predicts that total adenosine concentration rapidly saturates to a higher level (**Fig 2B**) and, on a slower timescale, total A_1_ receptor concentration progressively increases (**Fig 2D**), in response to the elevated adenosine concentration. Under chronic sleep restriction, there is a smaller increase in total adenosine concentration (**Fig 2B**). The progressive increase in total A_1_ receptor concentration is thus slower, occurring at about half the rate as it does under acute sleep deprivation (**Fig 2D**). The overall effect of each condition on sleep homeostatic pressure and cognitive performance can be assessed by examining the concentration of bound A_1_ receptors (**Fig 2C**). This reveals that 8 days of chronic sleep restriction causes a similar cognitive impairment to 1–2 days of acute sleep deprivation, in line with experimental results [4,5]. However, it takes about 4 days of acute sleep deprivation to generate the same up-regulation in total A_1_ receptor concentration as does 8 days of chronic sleep restriction.

### Cognitive performance

Within the physiological constraints discussed in Materials and Methods, the model achieves a good fit to the PVT data from Experiment 1, with an adjusted R^2^ value of 0.66. The same adjusted R^2^ value was obtained both with the initial fit to Experiment 1 and with the final (recalibrated) parameter values. Values for fit parameters are in **Table 1**. Notably, values for *K*_*d*1_ and *K*_*d*2_ are both at the lower end of the allowed (physiological) range. This suggested that a better fit may exist outside of the range. Relaxing the lower bounds on *K*_*d*1_ and *K*_*d*2_, a best fit was achieved at *K*_*d*1_ = 0.011 nM and *K*_*d*2_ = 5.0 nM, with a slightly better adjusted R^2^ value of 0.73, but this solution was discarded on the grounds of physiological constraints. The system’s ability to capture PVT performance to similar accuracy both inside and outside the empirically-observed ranges for *K*_*d*1_ and *K*_*d*2_ suggests that these ranges exist due to other biological constraints.

**Fig 3:**
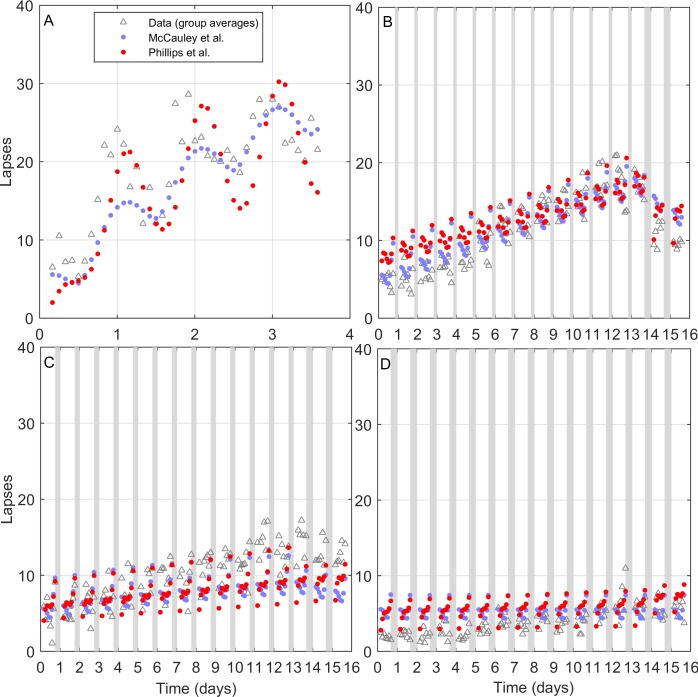
PVT data (open triangles) and simulations (filled circles) for Experiment 1, with time in bed (TIB) illustrated as gray bars. Panels show four experimental conditions: (A) Acute sleep deprivation with 0 h TIB, (B) 4 h TIB for 13 nights, then 2 nights of 8 h TIB, (C) 6 h TIB for 13 nights, then 2 nights of 8 h TIB, (D) 8 h TIB for 15 nights. PVT data were collected every 2 h during wakefulness, beginning 4 h after morning awakening, which was simulated to occur at 8am in all conditions (schedules from experimental data were shifted by 0.5 h for convenience). Zero time on the x-axis corresponds to midnight on the last baseline night. Data points are adapted from [17].

Fits to each of the experimental conditions are shown in **Fig 3**. The model performs especially well in fitting the 4-h time in bed and 8-h time in bed conditions. Some minor discrepancies between model and data are also observed. Under acute sleep deprivation, there is a slight mismatch in circadian phase; this is likely due to (i) the model fitting an average circadian phase to all conditions, (ii) drift away from a period of 24 hours under constant conditions, and (iii) data being restricted to certain circadian phases in the other three conditions. There is also a tendency for the model to underestimate PVT lapses in the 6-h time in bed condition. This same issue was faced by McCauley et al. when they fit their model to the same dataset; they attributed this to one outlier participant in the 6-h group who was unusually sensitive to the effects of sleep restriction [17]. The predictions of the McCauley et al. model are shown in **Fig 3** for reference, since our model’s parameters were fit to the exact same dataset. In general, the models closely agree under these simulated conditions. Both models predict a characteristic within-day variation in performance, with a sudden decline in performance in the final hours of awakening. This corresponds to the onset of the circadian night, as the circadian phase of maximal alertness is passed and homeostatic sleep pressure continues to build. This is consistent with dependence of PVT performance on circadian phase [3]. For PVT data, we find adjusted R^2^ = 0.66. Using the McCauley et al. model, which has a similar number of total parameters and no explicit physiological constraints, we find adjusted R^2^ = 0.70 on the same data set.

### Sleep

The same model used to simulate PVT lapses in Experiment 1 can also account for changes in sleep duration and timing during recovery from chronic sleep restriction in Experiment 2. Depending on the value of *λ* and the separation between the thresholds *D*_sleep_ and *D*_wake_, the model was found during the fitting procedure to generate a variety of different sleep patterns, from one sleep bout per night to multiple sleep bouts per night. Smaller values of *λ* and smaller separations between the thresholds favored more sleep bouts per night, due to the shorter time required to transit between thresholds. This finding is consistent with previous results found in the two-process model [6] and physiological models of mammalian sleep [26,42].

**Fig 4:**
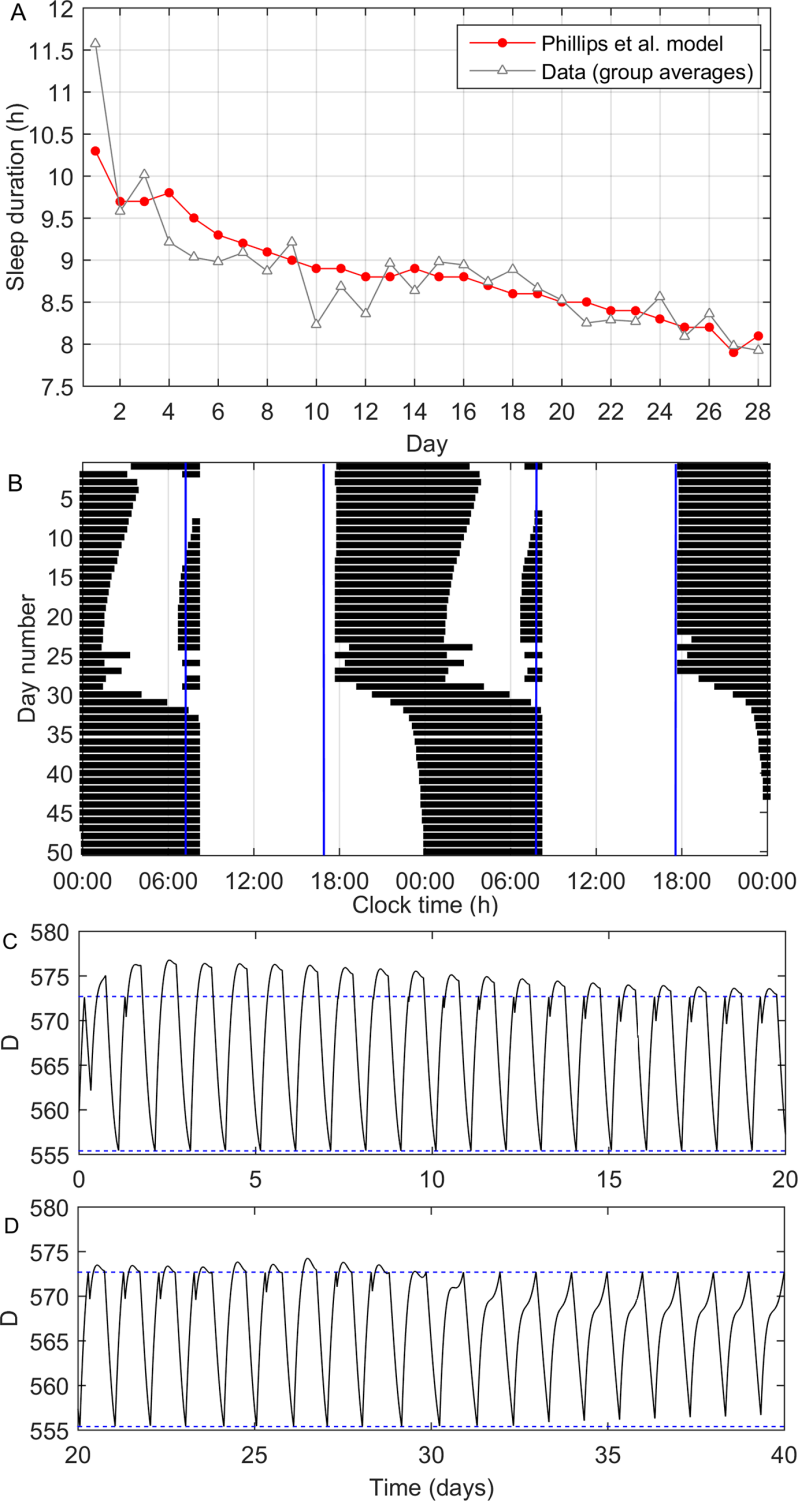
Daily sleep durations and sleep patterns during recovery from sleep chronically restricted to 7 h per night, with sleep allowed for 14 h per day from 18:00 to 8:00. (A) Daily sleep durations over 28 days for experimental data [40] and the model’s best fit. (B) Sleep patterns exhibited by the model across 50 days, displayed as a raster diagram, with black bars corresponding to sleep. The data are double plotted. During different stages of recovery, the model predicts both monophasic sleep (one sleep episode per day) and biphasic sleep (two sleep episodes per day). Vertical blue lines indicate the start and end of allowed sleep times. (C) and (D) show the sleep drive as a function of time across days 1–20 and 21–40 respectively, with the blue dashed lines representing the upper and lower sleep/wake thresholds.

**Fig 4** shows the model’s optimal fit to Experiment 2. In general, the model and data closely agree, with a root mean square error of 0.36 h. The model underestimates sleep duration on the first night by 1.3 h, then is within 0.7 h of the experimental data on all subsequent nights. During the recovery process, the model exhibits both monophasic sleep (one sleep bout per night), and biphasic sleep (two sleep bouts per night). This is interesting, since both sleep patterns were experimentally observed in different participants in Experiment 2. Some participants consistently had one sleep bout throughout the experiment, others consistently had two sleep bouts, while others alternated between one and two sleep bouts on different nights [40]. This suggests that the human population may span the region of parameter space that encompasses these two different modes of sleeping, in agreement with evidence that some humans historically had a split sleep pattern [41]. We note, however, that the model’s biphasic sleep patterns in **Fig 4** are a transient response to long nights, following a period of insufficient sleep. After approximately 30 days, sleep timing spontaneously shifts considerably later and consolidates back into a monophasic pattern (**Fig 4B**) as the system returns closer to the well-rested equilibrium state. This prediction cannot be compared to data, since the experiment ended after 28 days. The observed sleep/wake patterns can be better understood by observing the change in total sleep drive in **Fig 4C** and **4D**. During most of days 1–20, when sleep pressure remains relatively high, the main sleep bout occurs early and there is time after the main sleep bout for the sleep drive to reach the upper threshold again, resulting in a second sleep bout. This results in a spike and wave shape for D. Later, when sleep pressure is relatively dissipated, the main sleep bout occurs later and there is no longer time for a second sleep bout (i.e., sleep becomes monophasic).

## Discussion

The adenosine system has been proposed as a putative mechanism for changes in cognitive performance and sleep with sleep restriction [43,44]. In this paper, we developed a physiologically-based model of the brain’s adenosine system and showed that its dynamics capture changes in cognitive performance and sleep during acute sleep deprivation, chronic sleep restriction, and recovery from chronic sleep restriction.

**Fig 5:**
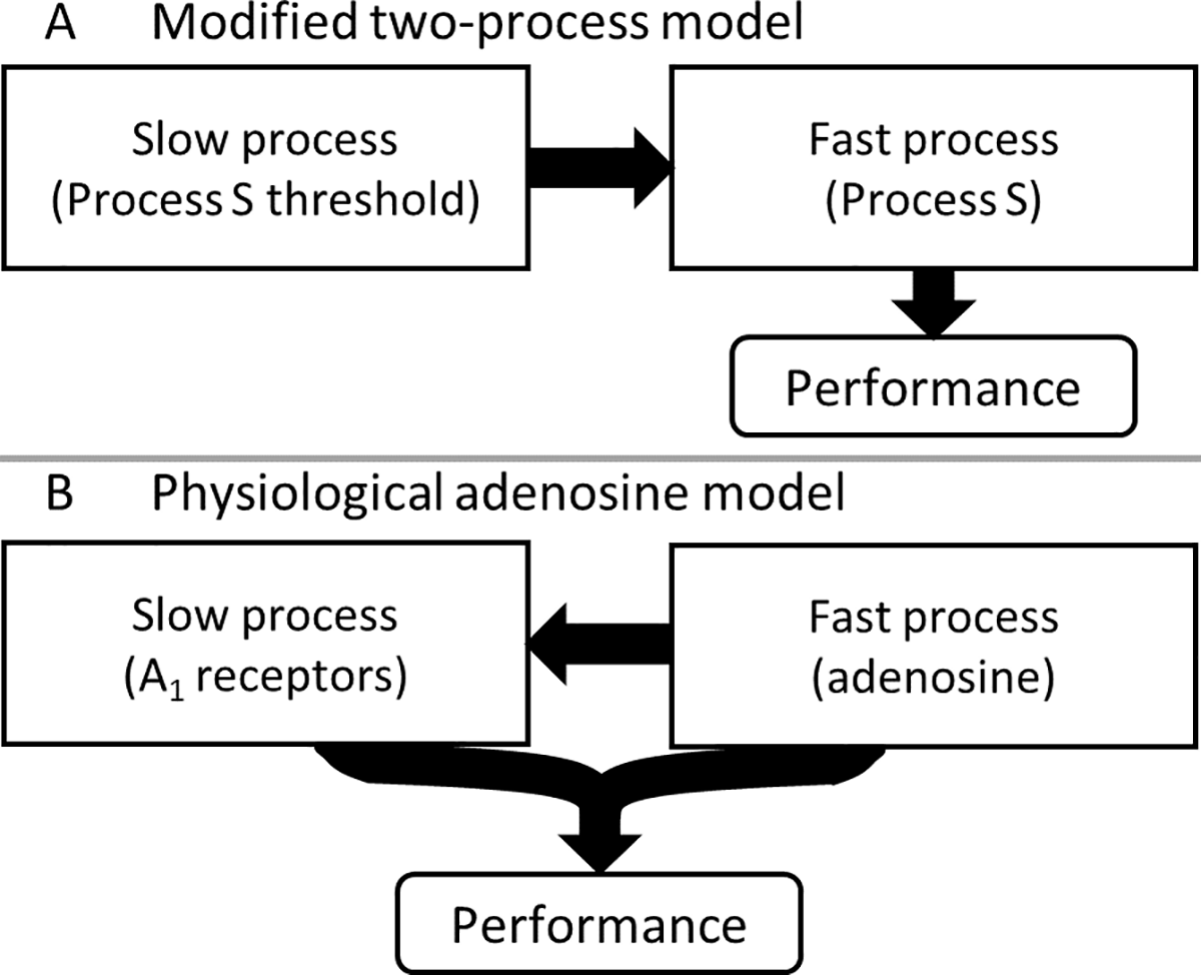
Mathematical structure of (A) previous mathematical models based on the two-process model, and (B) the physiologically based adenosine model developed here. Arrows show functional dependences between model variables.

Our physiological model performs similarly well to the best existing phenomenological models, which are based on the two-process model. Each of these models includes a fast homeostatic variable and a slow homeostatic variable. It was previously proposed that the variables of two-process-based models could therefore represent adenosine concentration and adenosine receptor concentration [17]. If so, a variable transformation should link phenomenological models to a physiological model. In investigating this, however, we found that our model is structurally different from two-process-based models. As illustrated in **Fig 5**, all existing two-process-based models include a dependence of the fast variable (Process S) on the slow variable, since the slow variable modifies the asymptotic behavior of Process S. Cognitive performance is then assumed to be a function of the fast variable. While these fast and slow variables have previously been interpreted as possible elements of the adenosine system based on pharmacokinetic principles [17,22], this mathematical structure is notably distinct from our model, in which the slow variable (A_1_ receptor concentration) responds to the fast variable (adenosine concentration), and the fast variable’s dynamics are independent of the slow variable. Cognitive performance in our model is then dependent on bound A_1_ receptors, which functionally depends on both the fast and slow variables. Due to these differences in model structure, the models will differ in their predictions in certain settings, such as protocols involving alternating cycles of sleep restriction and recovery, as we recently demonstrated [45]. Finding and testing such conditions will therefore be important to distinguishing which model structures are closest to the underlying biology.

A further important mathematical distinction exists between our model and the prior McCauley et al. model. Whereas our model’s dynamics are always convergent, the McCauley et al. model’s predictions are divergent in the long-time limit for sleep durations shorter than a critical duration [17]. This feature of convergence improves the McCauley et al. model’s performance on schedules that involve very short sleep opportunities (<4 h), which is one condition in which our model should next be tested. In a physiological context, we expect dynamics to converge, since it is unclear how to physiologically interpret divergence. The PVT output of our model is also a bounded function (sigmoid) of the physiological variables, implying a ceiling for performance impairment, reflective of the fact that there is a theoretical limit on the number of possible lapses per test. We note that our model is similar in the long-time limit to a previous phenomenological approach that assumed a ceiling on lapse probability [46]; one difference being that our model output is total number of lapses per test rather than lapse probability per trial. While our model is designed to test a hypothesis related to the adenosine system, there are many other important sleep-promoting molecules in the brain. These include cytokines, nitric oxide, and prostaglandins. As more data come to light, it may be necessary to extend our model to include other ligand/receptor systems and potential interactions between these systems. A sensible next step would be modeling the dynamics of A_2A_ receptors, which are involved in mediating effects of caffeine [47]. Previously we developed a physiological model of the neuronal systems that regulate sleep and alertness [32], including the effects of caffeine on subjective alertness and sleep [48]. The effects of caffeine were also recently included in another model of cognitive performance [49]. Both these models of caffeine, however, currently use a sleep homeostatic process that is not directly linked to adenosine receptor dynamics [50]. Integrating neuronal sleep models with the adenosine system model developed here is therefore an important future goal with multiple applications.

In experiments involving rodents, there is some evidence of an allostatic response to chronic sleep restriction, meaning the homeostatic set-point may change in response to chronic sleep restriction. Specifically, EEG slow-wave activity in male F344 rats is increased by sleep restriction on the first day, but this elevation disappears as sleep restriction continued on days 2–5. Moreover, when the animals recover, slow-wave activity falls below baseline levels [51]. Such a response could be explained by a decrease in the rate of adenosine production [52]. The existence of an allostatic response is, however, disputed. Leemburg et al. [53] found slow-wave activity was consistently elevated during both sleep restriction and recovery in male WHY rats, although this conflict could be due to strain differences. A decrease in slow-wave activity during chronic sleep restriction has not been reported in humans, but slow-wave activity notably shows little to no change during chronic sleep restriction, despite declining cognitive performance [4]. This outcome could be explained by different receptors mediating different functions (slow-wave activity vs. cognitive performance) and responding differently to sleep restriction. Indeed, there is evidence of A_2A_ receptor down-regulation in some brain regions following sleep deprivation [30]. This would be consistent with the finding that selective A_2A_ agonists promote non-rapid-eye-movement sleep, while selective A_1_ agonists do not [54]. However, there is significant functional overlap between receptor types, since A_1_ receptors also mediate slow-wave activity changes in response to sleep restriction [43], so it cannot be as simple as A_1_ receptors controlling cognitive performance and A_2A_ receptors controlling slow-wave activity; a more complex model is needed.

Our model has limitations and raises new questions. We have tested the model against two datasets, but in each case, we used group-average data, similar to other chronic sleep restriction models in their initial development [17,18]. Accounting for individual differences is particularly valuable, given large and stable differences between individuals in their vulnerability to sleep restriction on specific tasks [55,56]. Ramakrishnan et al. recently reported fitting a two-process-based model on an individual basis, but only after excluding the 3 most variable participants from a sample of 18 [57]. Since our model is based on underlying physiology, it could potentially be used to identify candidate mechanisms that account for individual differences. Ultimately, these mechanisms could be empirically tested using adenosine receptor imaging techniques [58].

In summary, we present the basis for a new model that is the first to explicitly and quantitatively link the sleep homeostatic process, cognitive performance, and sleep patterns to an underlying physiological and molecular biological mechanism. Although we are constrained by physiology in posing this model and fitting its parameter values, it performs similarly to the best existing phenomenological ad hoc models for PVT performance, which are free of such constraints. In addition, it advances beyond most of these models in accurately predicting sleep. The model could therefore be used to generate one-step predictions of both sleep and performance, rather than common two-step approaches in which sleep patterns are first derived and performance then predicted [59]. Our findings provide a physiologically plausible basis for observed changes in cognitive performance and sleep under a variety of experimental conditions. Critically, our model’s structure sheds light on the potential origin of empirical observations that sleep homeostasis involves dynamics on both short and long time scales.
